# Advanced MRI manifestations of trigeminal ganglioneuroma: a case report and literature review

**DOI:** 10.1186/s12885-016-2729-8

**Published:** 2016-08-30

**Authors:** Xiaojuan Deng, Jingqin Fang, Qingya Luo, Haipeng Tong, Weiguo Zhang

**Affiliations:** 1 Department of Radiology, Institute of Surgery Research, Daping Hospital, Third Military Medical University, 400042 Chongqing, China; 2 Department of Pathology, Institute of Surgery Research, Daping Hospital, Third Military Medical University, Chongqing, China; 3 State Key Laboratory of Trauma, Burns and Combined Injury, Institute of Surgery Research, Daping Hospital, Third Military Medical University, Chongqing, China

**Keywords:** Diffusion-weighted imaging, Diffusion tensor imaging, MR spectroscopy, magnetic resonance imaging, Trigeminal ganglioneuroma

## Abstract

**Background:**

Ganglioneuroma is a rare benign tumor originating from the sympathetic nerves, and its origination from the trigeminal nerves is even rarer. Only 4 cases of ganglioneuroma originating from the trigeminal nerve have previously been reported, and these studies only reported conventional MRI manifestations. To our knowledge, the advanced MRI features of trigeminal ganglioneuroma have not been reported thus far.

**Case presentation:**

This study reports a case of trigeminal ganglioneuroma in the left cerebellopontine angle. Advanced MRI showed the following tumor characteristics: significantly increased perfusion on perfusion imaging; isointense on diffusion-weighted imaging, whorled appearance within the tumor and no significant signs of damage to the white matter fiber tracts in the fractional anisotropy color map, and compare to the adjacent brain tissue, Choline didn’t show markedly elevation, and N-acetylaspartate peak showed slightly reduction on magnetic resonance spectroscopy. The tumor was completely resected, and the diagnosis of ganglioneuroma was confirmed by postoperative pathological examination.

**Conclusion:**

This case demonstrates the conventional as well as advanced MRI manifestations of this rare extra-axial tumor, which have never been previously reported. In addition, we reviewed the literature to demonstrate the advanced MRI features of trigeminal ganglioneuroma, in order to aid preoperative diagnosis and differentiation.

## Background

Ganglioneuromas are rare benign tumors originating from the ganglia and often arising in the retroperitoneum and posterior mediastinum. Usually, these tumors are discovered incidentally in adolescents or adults during thoracic or abdominal examinations [1–4]. Ganglioneuromas originating from the trigeminal nerves are even rarer, and to our knowledge, only 4 cases have been reported [5–8]. In all these cases, conventional MRI was conducted, and although its usefulness and role in tumor detection and evaluation cannot be denied, conventional MRI fails to provide information related to cellularity, tumor type, and grade, or even the true extent of the tumor. Therefore, in the current study, we report the advanced MRI characteristics of ganglioneuromas in the cerebellopontine angle, including characteristics observed on diffusion-weighted imaging (DWI), diffusion tensor imaging (DTI), susceptibility-weighted imaging (SWI), MR spectroscopy (MRS), and dynamic susceptibility-weighted contrast-enhanced perfusion (DSC perfusion). We also present a review of the literature to further clarify the characteristics of this rare tumor.

## Case presentation

**Fig. 1:**
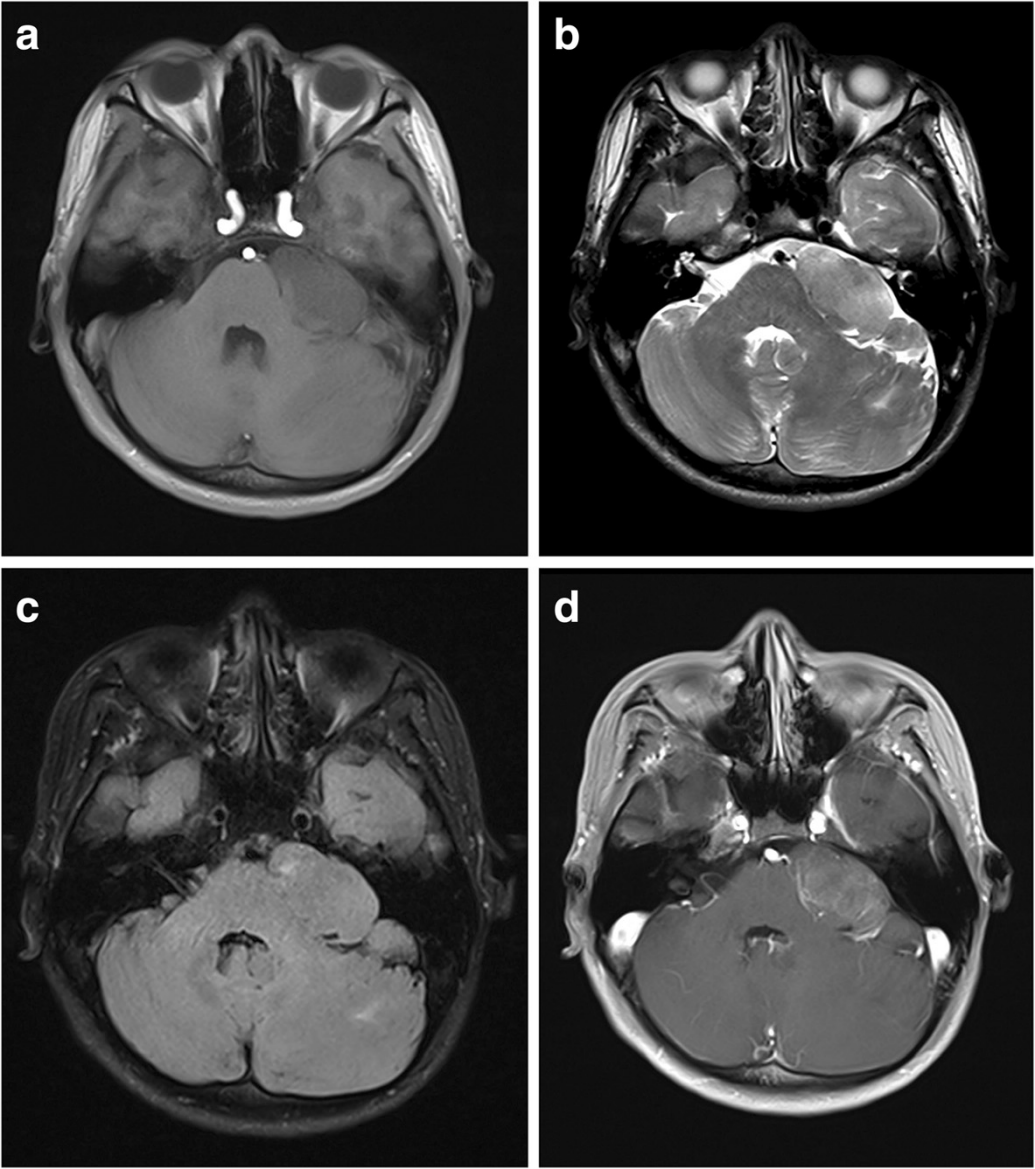
Conventional MRI of trigeminal ganglioneuroma. The tumor was homogeneously hypointense on T1WI (**a**),and mix iso-hyperintense on T2WI (**b**) and T2WI-Flair (**c**), it showed heterogeneous moderate enhancement on enhanced imaging (**d**)

The patient was a 19-year-old man who had been suffering from headaches and dizziness for 7 years prior to presentation, during which he complained of intermittent pain in the left zygomatic region. Neurological and laboratory investigations showed normal results. Brain MRI (Magnetom Verio 3.0T; Siemens, Germany) revealed a 4.4 cm × 3.6 cm × 2.5 cm well-circumscribed mass in the left cerebellopontine angle and mild compression of the left middle cerebellar peduncle and left cerebellar hemisphere. The tumor appeared homogeneously hypointense on T1WI (Fig. 1a), mix iso to hyperintense on T2WI (Fig. 1b) and T2WI-Flair (Fig. 1c), and showed heterogeneous moderate enhancement on contrast-enhanced imaging (Fig. 1d).

**Fig 2:**
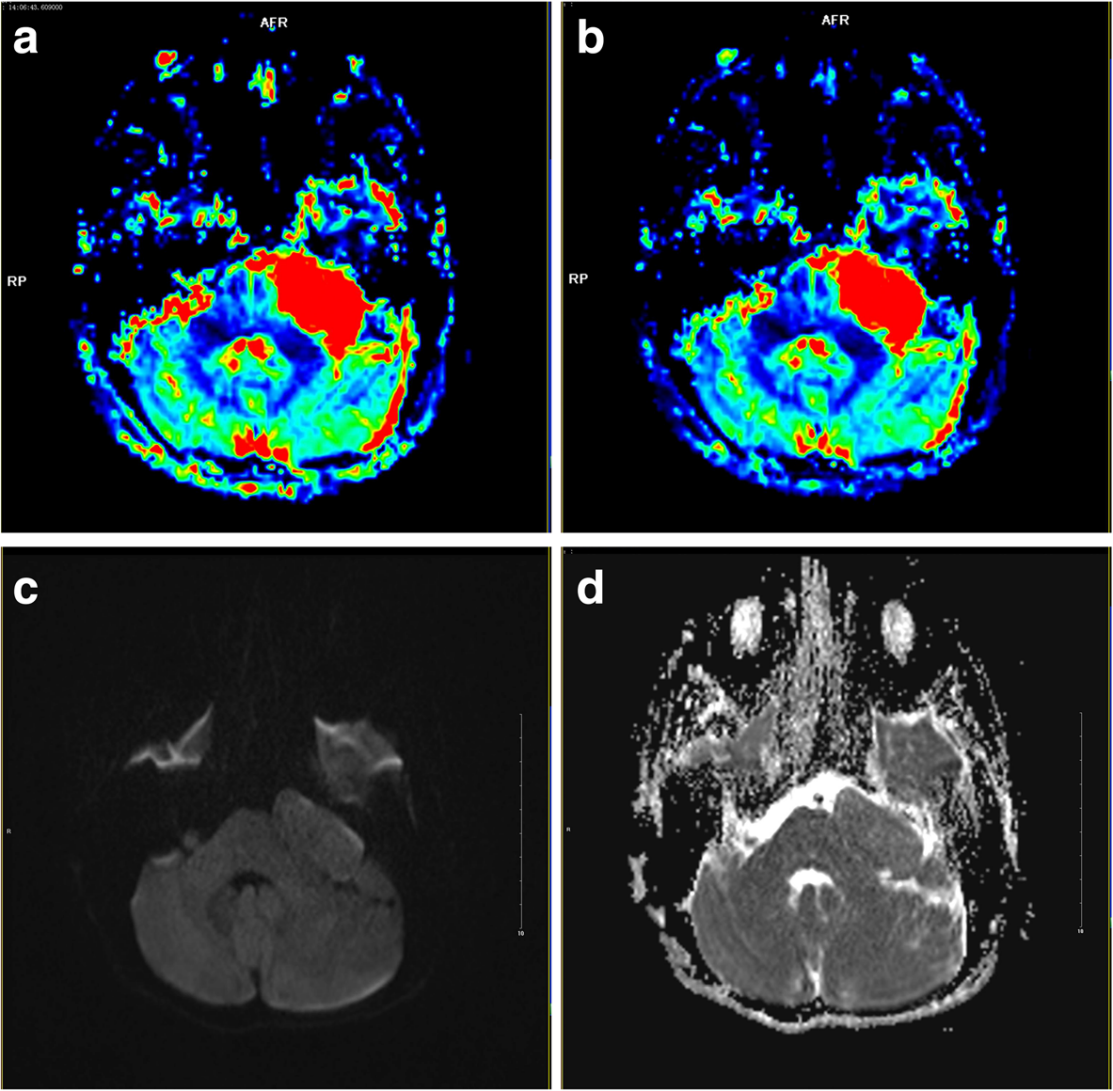
rCBV and rCBF on DSC perfusion, DWI and ADC map of trigeminal ganglioneuroma. The tumor showed increased rCBV (**a**) and rCBF (**b**), and homogeneous isointense on DWI (**c**). and higher ADC value on ADC map (**d**) compared with adjacent brain tissues

**Fig 3:**
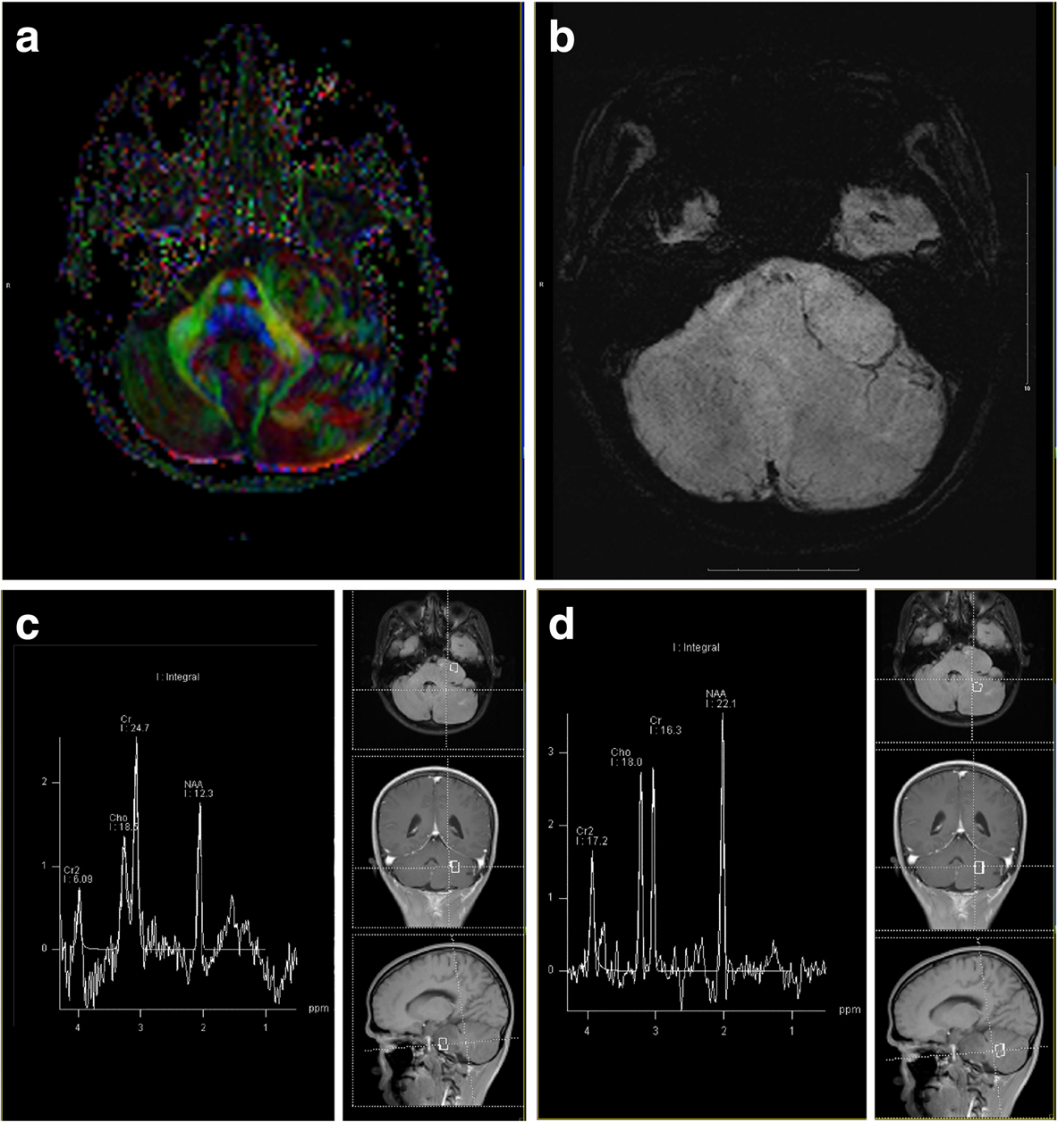
FA color map, SWI and single voxel proton MRS of trigeminal ganglioneuroma. **a** On the FA color map, the white matter fiber tracts surrounding the tumor appeared compressed and had shifted, but not damaged. A characteristic whorled appearance was showed in tumor. **b** Significant low signals suggesting bleeding or calcification were absence in the tumor on SWI. **c** Representative MRS imaging of tumor, left showed the MRS imaging, right showed the place of the voxel (square). **d** Representative MRS imaging of adjacent normal tissue, left showed the MRS imaging, right showed the voxel of adjacent normal tissue. Cho was not elevated and NAA peak was slightly reduced on proton MRS compare to adjacent normal tissues

On perfusion MRI, the tumor showed increased perfusion, and both relative cerebral blood volume (rCBV) (Fig. 2a) and relative cerebral blood flow (rCBF) (Fig. 2b) were elevated. The tumor appeared homogenously isointense on DWI (Fig. 2c). Apparent diffusion coefficients (ADCs) (Fig.2d) were measured in 5 non-overlapping circular regions of interest on tumor, CSF and adjacent brain parenchyma respectively, The mean ADC values of the tumor were 0.91 × 10^−3^ mm^2^/s, compared to 3.69 ×10^−3^ mm^2^/s for CSF (measured in the contralateral cerebellopontine angle) and 0.79 × 10^−3^ mm^2^/s for adjacent brain parenchyma (measured in the left cerebellum). A fractional anisotropy (FA) color map showed that although the adjacent white matter fiber tracts were not damaged, they had shifted because of pressure from the tumor. Additionally, the tumor showed a characteristic whorled appearance (Fig. 3a). On SWI, black low-signal were absence in the tumor, suggesting the absence of bleeding or calcification within the tumor (Fig. 3b). On single-voxel proton MRS, the tumor region (Fig. 3c) didn’t show markedly elevated choline (Cho),but show slightly lower levels of N-acetylaspartate (NAA), and Cho/creatine (Cr) and NAA/Cr, and a slightly higher Cho/NAA ratio compare to adjacent cerebellum (Fig. 3d) (Table 1).

**Table 1:** Parameters on MRS and ADC value on DWI

	Cho	NAA	Cr	Cho/Cr	NAA/Cr	Cho/NAA	ADC value
Tumor	18.5±3.1	16±3.3	24.7±0.7	0.75±0.2	0.65±0.3	1.16±0.3	0.91±0.16
Normal	18.0±1.4	22.1±0.6	16.3±1.8	1.10±0.3	1.36±0.4	0.81±0.5	0.79±0.21

**Fig 4:**
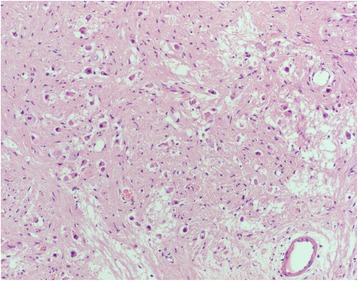
Histopathological examination (HE ×100) showed a benign tumor composed of abundant collagen stroma and spindle-shaped cells, and containing clustered mature ganglion cells in it

On the basis of imaging findings and lesion location, the patient was preoperatively diagnosed with a neurogenic tumor. He underwent left suboccipital craniotomy and tumor excision. Pathological examination showed that the tumor was solid and well-circumscribed and closely adhered to the trigeminal nerve. HE staining (Fig. 4) showed that the tumor comprised abundant collagen stroma and spindle-shaped cells, along with clustered mature ganglion cells. Immunohistochemical analysis showed S-100 (+), NeuN (+), GFAP (+), NF (+), and a Ki-67 of approximately 1%, which confirmed a pathological diagnosis of ganglioneuroma.

## Discussion

Ganglioneuroma is a rare benign tumor of the sympathetic nerves originating from the peripheral nervous system, and it commonly occurs in the retroperitoneum and posterior mediastinum, fewer cases are reported in head [9]. Intracranial ganglioneuroma is extremely rare, and only 4 cases of ganglioneuroma originating from the trigeminal nerve have been reported [5–8].

Thus, the imaging manifestations of ganglioneuroma originating from the trigeminal nerve have rarely been reported. In the present study, we reviewed 5 cases of ganglioneuroma originating from the trigeminal nerve [5–8], including the present case, and summarized the observed conventional MRI manifestations (Table 2). The typical sites of origin are the cerebellopontine angle and middle cranial fossa, a finding consistent with the tracking of the trigeminal nerve. The largest diameter of the tumor ranged from 2.0 to 4.5 cm (mean, 3.5 cm), and the tumors presented as well-circumscribed oval lesions, with iso-hypointense on T1WI and hyperintense or mix iso-hyperintense on T2WI and T2WI-Flair. Enhanced MRI showed slight, moderate, or obvious enhancements and most were heterogeneous. The signal patterns of trigeminal ganglioneuromas are consistent with those of ganglioneuromas arising at other common locations [1–4]. Conventional MRI alone, however, might fail to distinguish trigeminal ganglioneuromas from other extra-axial tumors or tumor-like lesions, because it does not provide information on cellularity, tumor type, grade or the actual tumor extent.

**Table 2:** Patient characteristics (*n*=5) of trigeminal ganglioneuroma case reports

Patient	Gender	Age	Location	Diameter (cm)	Border	T1WI	T2WI	T2WI-Flair	Enhancement	DWI
1	F	8	C-P angle		Clarity	Hypointence	Hyperintence		Obvious heterogeneous	
2	F	60	Middle cranial fossa	4.5	Clarity	Hypointence	Hyperintence	hyperintence	Obvious heterogeneous	
3	M	55	Middle cranial fossa	3.0	Clarity	Mix-signals	Mix-signals		Obvious heterogeneous	
4	F	44	C-P angle	2	Clarity	Isointense	Hyperintence		Slight heterogeneous	Hyperintense
5	M	19	C-P angle	4.4	Clarity	Hypointence	Mix iso-hyperintence	Mix iso-hyperintence	Moderate heterogeneous	Isointense

The data of case 1–4 come from the references of 5–8

Advanced MRI provides more information related to the histological features and physiological metabolic characteristics of tumors, such as angiogenesis classification, cellularity, and mitotic indices. In the present study, the advanced MRI manifestations of the trigeminal ganglioneuroma were analyzed, including DWI. ADC values, FA color map, MRS, DSC perfusion, and SWI manifestations. In addition, the literature was reviewed to further clarify the advanced MRI characteristics of these tumors.

DWI represents the cell concentration of the tumor; wherein a hyperintense signal indicates high cell concentration. The ADC value represents the mobility of free water molecules in the tissues, and a low ADC value indicates relatively high cell density. In the present case, we observed an isointense signal on DWI and a higher mean ADC value (0.91 × 10^−3^ mm^2^/s) than the adjacent brain tissues (0.79 × 10^−3^ mm^2^/s). Further, the ADC value was higher than that in the study reported by Kim et al. [8] (0.72 × 10^−3^ mm^2^/s). Gahr et al. [10] also observed that the mean ADC value of ganglioneuroma/ganglioneuroblastoma was significantly higher than that of neuroblastoma. These features are corresponded to the histopathological characteristics of a ganglioneuroma, such as abundant myxoid stroma and relatively few cellular components on microscopic examination [9, 11], and collectively suggest that the tumor shows the histological characteristics of low-grade tumors rather than high-density or high-grade tumors.

The DTI technique represents the anisotropic diffusion of water molecules and is used to track white matter fiber tracts. It is useful for determining the position of these tracts and studying tumor effects (such as the displacement of nervous fiber tracts or significant damage due to tumor invasion) [12, 13]. In the current case, the FA color map showed that the left middle cerebellar peduncle was compressed by the tumor, leading to consequent compression, but no distruction of white matter fiber tracts. These are confirmed manifestations of benign extra-axial tumors. Some studies have reported that the whorled appearance is one of the characteristic MR findings for diagnosing ganglioneuroma [1, 3, 9], but the features have not been demonstrated in these 5 cases on conventional MRI. The whorled appearance of the tumor which showed on FA map might represent the tracking features of the spindle-shaped cells and it has not been observed in other brain tumors [14]. Hence, the whorled appearance on the FA map could be useful to differentiate trigeminal ganglioneuromas from other tumors.

Proton MRS is a valuable technique for the differential diagnosis of high- and low-grade tumors. Cho represents metabolism in the cell membrane, including the production and repair of myelin, and elevated levels of Cho represents cell proliferation or increased metabolism [15]. In the current case, the Cho/Cr ratio (0.75) was significantly lower than that in high-grade (2.43~1.7) and low-grade (1.75~1.2) gliomas reported previously [16, 17], suggesting less cell proliferation in the current tumor, which is consistent with the histopathological characteristics of low-grade tumors. On MRS, the NAA peak is a specific marker of neuronal viability, and it may indicate the presence of neurons which are cell markers of a pathological diagnosis of ganglioneuroma, in extra-axial tumors.

Perfusion MRI accurately reflects the grade of gliomas. The rCBV is reliably correlated with glioma grade and the histological findings of increased tumor vascularity [17, 18]. In the current case, the rCBV and rCBF were both high, indicating increased perfusion, however, the histopathological findings in the present case revealed a low-grade tumor. Owing to the lack of a blood–brain barrier, most extra-axial hypervascular tumors have increased perfusion rates [19, 20], so perfusion MRI cannot be used for the differential diagnosis of extra-axial tumors.

SWI sequences are very sensitive to bleeding and calcification and show low signal intensity in the presence of these artifacts. In the present case, the tumor did not show significantly low signals, suggesting the absence of significant bleeding and calcification, which was consistent with the histological findings.

In the present case, the isointense signal on DWI and the relatively high ADC value indicated low cell concentration, on the FA color map, the compression but no destruction of adjacent white matter fiber tracts indicated benign extra-axial tumor, and the whorled appearance in the tumor represented the tracking of the spindle-shaped cells, and the lower Cho/Cr ratio and an NAA peak on MRS of such an extra-axial tumor indicated low cell proliferation and the presence of neurons. All these features which cannot be showed on conventional MRI,were consistent with the histopathological features of trigeminal ganglioneuroma, and indicated a low grade tumor. However, on DSC perfusion and SWI, this tumor did not show the characteristic features of trigeminal ganglioneuroma, because most extra-axial tumors show increased perfusion due to complete absence of a blood–brain barrier, and the present tumor showed no significant calcification or bleeding. From our findings, we concluded that a combination of several advanced MRI techniques might enable accurate preoperative diagnosis of trigeminal ganglioneuromas.

## Conclusions

The present case demonstrated the conventional and advanced MRI manifestations of the rare extra-axial tumor trigeminal ganglioneuroma, which have not been previously described in such detail. In addition, a review of the literature is presented to better understand the advanced MRI characteristics of trigeminal ganglioneuroma and provide more information related to the histological features and physiological metabolic characteristics of this tumor. We believe that advanced MRI is useful for the preoperative diagnosis and differentiation of trigeminal ganglioneuroma from other tumors and tumor-like lesions. However, further large-scale studies are warranted to confirm these characteristics.

## Consent

This study was approved ethically by Institute of Surgery Research, Daping Hospital, Third Military Medical University (Chongqing, China) (Approval ID: [2015]23) and written informed consent was obtained from the patient for publication of this case report and any accompanying images. A copy of the written consent is available for review by the Editor of the journal.

## Abbreviations

ADC: Apparent diffusion coefficient
Cho: Choline
DSC perfusion: Dynamic susceptibility-weighted contrast-enhanced perfusion
DTI: Diffusion tensor imaging
DWI: Diffusion-weighted imaging
FA color map: Fractional anisotropy color map
GFAP: Glial fibrillary acidic protein
MRI: Magnetic resonance imaging
MRS: Magnetic resonance spectroscopy
NAA: N-acetylaspartate
NeuN: Neuron-specific nuclear protein
NF: Neurofilament protein
rCBF: Relative cerebral blood flow
rCBV: Relative cerebral blood volume
SWI: Susceptibility-weighted imaging
